# Shorter Versus Prolonged Systemic Perioperative Antibiotic Prophylaxis in Clean Orthopedic Surgery: A Systematic Review and Meta-Analysis

**DOI:** 10.3390/antibiotics15080759

**Published:** 2026-08-07

**Authors:** Mohamedanas Mohamedfaruk Patni, Ibrahim Alabid, Malak Abedi, Mohamed El-Tanani, Imran Rangraze, Syed Arman Rabbani, Rasha Aziz Attia Salama, Khaled Walid Elayyan, Aiswarya Menon

**Affiliations:** 1 Department of Community Medicine, RAK College of Medical Sciences, RAK Medical and Health Sciences University, Ras Al Khaimah 11172, United Arab Emirates; rasha.aziz@rakmhsu.ac.ae; 2 RAK College of Medical Sciences, RAK Medical and Health Sciences University, Ras Al Khaimah 11172, United Arab Emirates; malak.abedi0320@gmail.com (M.A.); khaled.22901106@rakmhsu.ac.ae (K.W.E.); menonaiswaryais@gmail.com (A.M.); 3 RAK College of Pharmacy, RAK Medical and Health Science University, Ras Al Khaimah 11172, United Arab Emirates; eltanani@rakmhsu.ac.ae (M.E.-T.); arman@rakmhsu.ac.ae (S.A.R.); 4 Department of Internal medicine, RAK College of Medical Sciences, RAK Medical and Health Sciences University, Ras Al Khaimah 11172, United Arab Emirates; imranrashid@rakmhsu.ac.ae

**Keywords:** antibiotic prophylaxis, antimicrobial stewardship, orthopedic surgery, surgical-site infection, postoperative antibiotics, prophylaxis duration

## Abstract

**Background and Objectives:** Surgical-site infection remains a major complication of clean orthopedic surgery, particularly when implants or instrumentation are used. Although shorter perioperative prophylaxis is generally accepted for most clean procedures, prolonged postoperative courses continue to be used in selected high-risk orthopaedic settings. This review evaluated whether comparative evidence supports such extension. **Methods:** PubMed/MEDLINE, Scopus, and Cochrane CENTRAL were searched from inception to 14 May 2026. Randomized trials and non-randomized comparative studies evaluating systemic prophylaxis duration were eligible for qualitative synthesis. The primary meta-analysis was restricted to randomized controlled trials with extractable surgical-site infection data. Risk ratios were pooled using a random-effects model. Subgroup, sensitivity, and publication-bias analyses were performed. **Results:** Thirty-six studies were included in the qualitative synthesis, and 11 randomized trials involving 3823 participants contributed to the primary meta-analysis. After incorporating the cluster-adjusted estimate from the cluster-randomized trial, prolonged prophylaxis did not demonstrate a statistically significant additional reduction in any surgical-site infection compared with shorter prophylaxis (RR = 1.07, 95% CI 0.86–1.34; prediction interval 0.83–1.38; I^2^ = 0.0%). A sensitivity analysis excluding the cluster-randomized trial produced a similar result (RR = 1.11, 95% CI 0.88–1.40). Exploratory procedure-based subgroup analyses did not identify a statistically significant interaction, although each subgroup contained few trials. Secondary analyses showed no statistically significant difference for superficial or non-deep infection (RR = 1.19, 95% CI 0.79–1.77) or deep or complicated infection (RR = 0.85, 95% CI 0.55–1.32). **Conclusions:** Prolonged systemic prophylaxis has not demonstrated a consistent additional surgical-site infection-prevention benefit compared with shorter regimens. However, sparse events, wide confidence intervals, clinical heterogeneity, and low certainty preclude conclusions of equivalence or noninferiority. Further adequately powered trials are needed in higher-risk procedures.

## 1. Introduction

Surgical-site infection (SSI) remains one of the most important complications after orthopedic surgery, particularly when prosthetic joints, internal fixation devices, tumor endoprostheses, or spinal instrumentation are implanted. Although clean orthopedic procedures are performed without active infection or gross contamination, the introduction of foreign material increases the clinical consequence of bacterial contamination because infection may become implant-associated, persistent, and difficult to eradicate. Once established, infection can require prolonged antimicrobial therapy, surgical debridement, revision surgery, implant removal, or complex reconstruction, making prevention a central priority in orthopedic care [1,2].

Systemic perioperative antibiotic prophylaxis is a key component of SSI prevention, but its effectiveness depends on more than simply administering antibiotics. Appropriate agent selection, correct dosing, timely administration before incision, intraoperative redosing when indicated, and rational postoperative discontinuation all contribute to prophylactic quality. Evidence evaluating antibiotic choice and timing has shown that these factors can influence infection outcomes, meaning that the question of duration should be considered only after the initial prophylactic regimen has been optimized [3,4].

The principle of limiting unnecessary postoperative antibiotic exposure is well established in surgical prophylaxis practice. Nevertheless, prolonged courses continue to be used in selected orthopaedic settings, particularly when implants, spinal instrumentation, postoperative drains, tumour reconstruction, or other perceived high-risk features are present. The unresolved question addressed by this review is therefore not whether prolonged prophylaxis should be used routinely, but whether selected higher-risk orthopaedic procedures derive additional benefit from extension beyond shorter perioperative regimens. Randomized trials have examined this question across hip surgery, spine surgery, fracture fixation, arthroplasty, mixed clean orthopaedic surgery, and orthopaedic oncology, but have been limited by low SSI event rates, procedure-specific populations, varying antibiotic regimens, heterogeneous SSI definitions, and different follow-up periods [1,2,5,6,7,8,9,10,11,12].

This uncertainty is further complicated by differences between routine clean orthopedic procedures and higher-risk operations. Instrumented spine surgery, fracture fixation, arthroplasty, and tumor endoprosthetic reconstruction do not share the same baseline infection risk or operative complexity. Some trials enrolled low-event arthroplasty or hip-surgery populations, while others included higher-risk settings such as instrumented spinal procedures or lower-extremity tumor reconstruction [9,10,11,12]. These differences make pooled synthesis useful, but they also require careful interpretation of subgroup findings rather than assuming that all clean orthopedic procedures respond similarly to prolonged prophylaxis.

Nonrandomized studies have added important real-world context but have not resolved the question. Some cohort studies suggest that extended postoperative or oral prophylaxis may reduce infection in selected high-risk arthroplasty or spine populations, whereas other large database and institutional studies have not shown a consistent reduction in SSI or periprosthetic joint infection with prolonged prophylaxis [13,14,15,16]. These conflicting findings may reflect differences in patient selection, surgical complexity, local protocols, and confounding by indication, because extended antibiotics are often prescribed to patients already perceived as being at higher infection risk.

The possible benefit of prolonged prophylaxis must also be balanced against antimicrobial-related harms. Longer postoperative antibiotic exposure may increase the risk of adverse drug reactions, acute kidney injury, *Clostridioides difficile* infection, antimicrobial resistance, and healthcare costs. Large surgical cohorts and orthopedic-specific studies have raised concerns that broader or longer prophylaxis may increase patient harm without clearly improving infection prevention [17,18,19]. Therefore, the duration question has direct implications for antimicrobial stewardship as well as surgical outcomes.

A clinically useful synthesis must distinguish duration-comparison evidence from related but separate questions such as antibiotic choice, timing, and prophylaxis versus placebo. Studies of vancomycin-based regimens, cefazolin alternatives, pre-incision timing, or placebo-controlled prophylaxis provide important background, but they do not directly answer whether antibiotics should be continued after an appropriate perioperative regimen has already been given [20,21]. Separating these evidence categories is essential to avoid attributing the effects of agent selection or timing to duration itself.

Accordingly, this systematic review and meta-analysis aimed to compare shorter versus prolonged systemic perioperative antibiotic prophylaxis in clean orthopedic surgery. The primary outcome was any SSI, while secondary outcomes included superficial SSI, deep or complicated infection, periprosthetic joint infection, infection requiring reoperation or implant removal, antibiotic-related adverse events, acute kidney injury, *C. difficile* infection, antimicrobial resistance, readmission, reoperation, and mortality. The quantitative synthesis focused on randomized duration-comparison trials, while nonrandomized studies were synthesized qualitatively to contextualize real-world practice, safety, resistance, external validity, and procedure-specific uncertainty.

## 2. Materials and Methods

### 2.1. Protocol and Registration

This systematic review and meta-analysis was conducted and reported in accordance with the Preferred Reporting Items for Systematic Reviews and Meta-Analyses 2020 statement. The review protocol was registered in PROSPERO under registration number CRD420261423310. The review was designed to evaluate whether shorter systemic perioperative antibiotic prophylaxis differs from prolonged systemic prophylaxis in preventing surgical-site infection after clean orthopedic surgery.

Randomized controlled trials formed the primary evidence base for the quantitative meta-analysis. Nonrandomized comparative studies that evaluated systemic prophylaxis duration were included in the qualitative synthesis to contextualize the randomized findings, external validity, safety outcomes, resistance concerns, and real-world prescribing practice. Nonrandomized studies were not pooled with randomized trials.

### 2.2. Eligibility Criteria

Eligibility was defined according to the population, intervention, comparator, outcomes, and study design framework. Eligible populations included patients of any age undergoing clean orthopedic surgery, defined as surgery performed in the absence of active infection, gross contamination, septic revision, or an open contaminated traumatic wound. Eligible procedures included spinal surgery, primary joint arthroplasty, hip surgery, fixation of closed fractures, clean implant-related procedures, and lower-extremity bone-tumor resection followed by endoprosthetic reconstruction. Studies with mixed surgical populations were eligible only when data for clean orthopedic procedures were extractable or clearly relevant to the review question.

The intervention of interest was a shorter systemic perioperative antibiotic prophylaxis regimen. This included a single preoperative or perioperative dose, no postoperative continuation, fewer postoperative doses, discontinuation within 24 h, or the shorter regimen as defined by each comparative study. The comparator was a prolonged systemic prophylaxis regimen, including additional postoperative doses, continuation beyond 24 h, fixed 48-h or 72-h courses, multiday prophylaxis, continuation until drain removal, or extended oral antibiotic prophylaxis after discharge.

Antibiotics initiated or continued because of suspected or confirmed postoperative infection were considered therapeutic treatment rather than prophylaxis. Such exposure was excluded from prophylaxis-duration comparisons whenever it could be distinguished from protocolized prophylaxis. Studies primarily evaluating treatment of established infection were not eligible for the comparative prophylaxis analysis.

The primary outcome was any surgical-site infection, as defined by the investigators of each study. Secondary outcomes included superficial incisional infection, deep or organ/space infection, periprosthetic joint infection, complicated infection, infection requiring reoperation or implant removal, antibiotic-related adverse events, acute kidney injury, *Clostridioides difficile* infection, antimicrobial resistance, non-surgical-site infection, hospital length of stay, readmission, reoperation, and mortality.

Individually randomized trials, cluster-randomized trials, prospective comparative studies, retrospective comparative studies, historical-control studies, before-and-after studies, and database or registry-based comparative studies were eligible for the qualitative systematic review when they evaluated prophylaxis duration. The primary meta-analysis was restricted to randomized trials with extractable treatment-arm data for surgical-site infection. Studies were excluded from the formal evidence synthesis if they evaluated only antibiotic agent selection, antibiotic dose, preoperative timing, prophylaxis versus placebo without a duration comparison, local antibiotic delivery without systemic-duration comparison, therapeutic antibiotics for established infection, infection epidemiology without comparative duration analysis, non-orthopedic procedures, or non-comparative designs. Such studies were retained only as contextual references when relevant to the Introduction or Discussion.

### 2.3. Information Sources and Search Strategy

A systematic search was conducted in PubMed/MEDLINE, Scopus, and the Cochrane Central Register of Controlled Trials from database inception to 14 May 2026. The search combined controlled vocabulary and free-text terms related to orthopedic surgery, spinal surgery, arthroplasty, fracture fixation, implant surgery, antimicrobial prophylaxis, perioperative antibiotics, postoperative antibiotics, prophylaxis duration, single-dose regimens, multiple-dose regimens, short-course prophylaxis, prolonged prophylaxis, and randomized trials. No restriction was applied according to publication date, participant age, or geographic location.

Reference lists of eligible studies and relevant review articles were screened manually. Forward-citation searching was performed in Scopus to identify additional eligible reports. The complete database-specific search strategies are provided in Supplementary Table S1.

### 2.4. Study Selection

All records identified through the search were imported into EndNote (Clarivate, Philadelphia, PA, USA), and duplicate records were removed electronically and subsequently checked manually. Two reviewers independently screened titles and abstracts against the predefined eligibility criteria. Reports considered potentially eligible by either reviewer were retrieved in full text and assessed independently.

**Figure 1:**
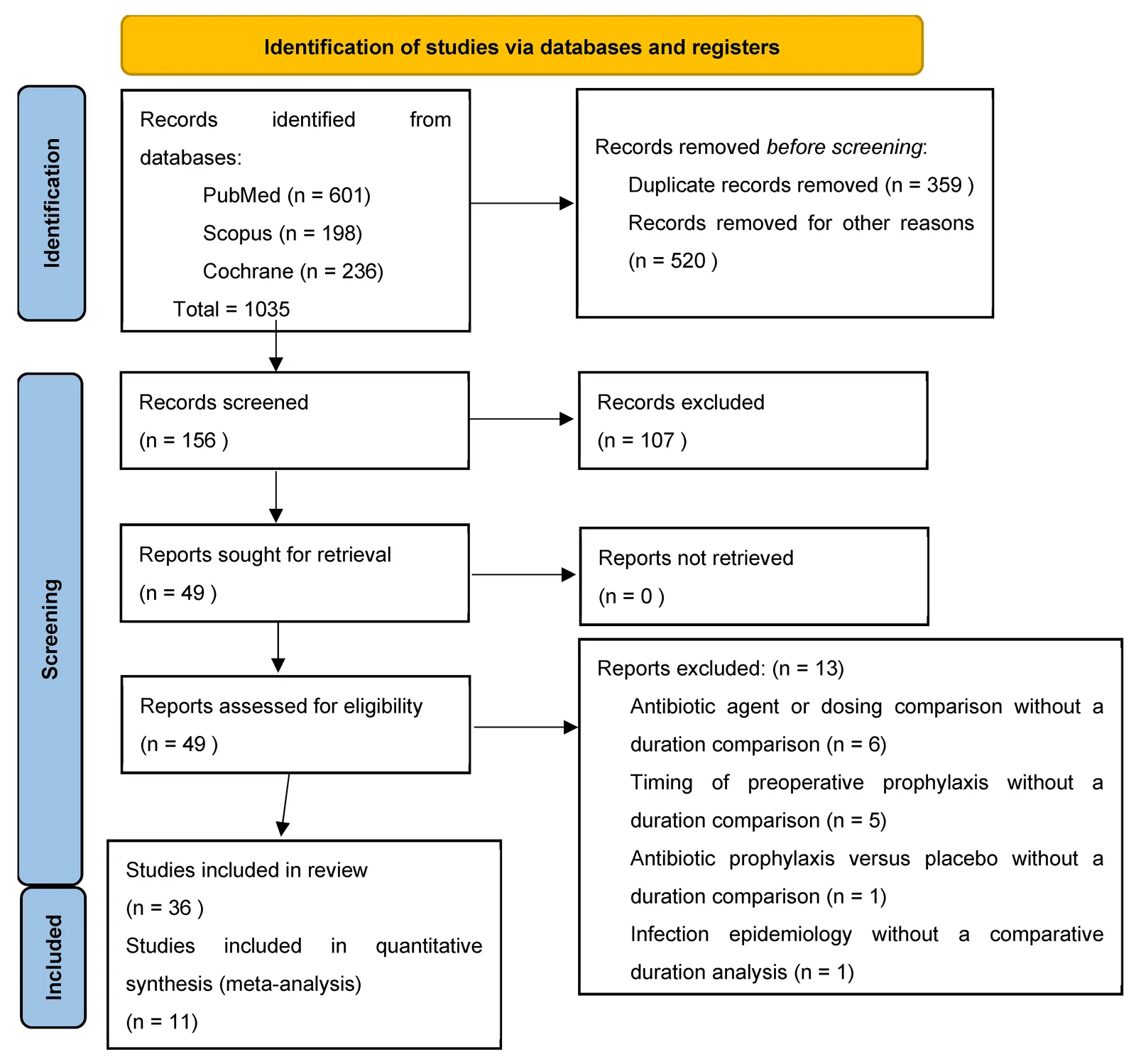
PRISMA flow diagram depicting the study selection process.

Disagreements at title/abstract or full-text stage were resolved through discussion, and a third reviewer was consulted when consensus could not be reached. Reasons for exclusion at full-text review were recorded using one principal exclusion reason per report. Multiple publications from the same study were linked and treated as a single study, with the most complete report used as the primary source of data. The complete study-identification and selection process, including database records, duplicate removal, title-and-abstract screening, full-text assessment, exclusion reasons, and final inclusion in the qualitative and quantitative syntheses, is summarized in the PRISMA flow diagram Figure 1.

### 2.5. Data Extraction

Two reviewers independently extracted data using a standardized extraction form. Extracted data were compared, and discrepancies were resolved by consensus or consultation with a third reviewer. Extracted data were recorded and managed using Microsoft Excel (Microsoft Corporation, Redmond, WA, USA). Data were extracted from the main article, tables, figures, Supplementary Material, and trial registration records where available.

The following information was extracted: first author, publication year, country, study design, number of centres, recruitment period, sample size, participant characteristics, surgical procedure, indication for surgery, implant or instrumentation use, drain use, antibiotic agent, dose, route, timing, intraoperative redosing, duration of prophylaxis in each group, postoperative oral antibiotic use, follow-up duration, surgical-site infection definition, treatment-arm event counts, adverse events, reoperation, readmission, antimicrobial resistance outcomes, funding source, and information required for risk-of-bias assessment.

For each randomized trial, we also extracted the maximum duration of follow-up, the surgical-site infection surveillance window, the time point selected for analysis, and whether outcome ascertainment extended beyond hospital discharge. Post-discharge surveillance methods were categorized as outpatient clinical review, telephone or postal follow-up, medical-record review, registry or administrative-data linkage, or another method reported by the investigators. We also recorded the SSI definition, infection depth, and whether outcomes were independently or blindly adjudicated. When the surveillance method, definition, or follow-up duration was not reported, the item was recorded as not reported rather than inferred.

We recorded whether postoperative antibiotic continuation represented protocolized prophylaxis or was initiated in response to postoperative clinical findings suggestive of infection. When the indication for continued antibiotics was unclear, this was recorded as a potential exposure-classification limitation.

For randomized trials, the number of participants and number of events in the shorter and prolonged prophylaxis groups were extracted for the primary surgical-site infection outcome. When several infection outcomes were reported, the study-defined overall surgical-site infection outcome was preferred. When overall infection was not reported, superficial and deep or complicated infection outcomes were extracted separately when available. For studies with incomplete or unclear treatment-arm outcome data, the study was retained in the qualitative synthesis but excluded from the affected quantitative analysis.

### 2.6. Outcome Definitions

The primary outcome was any surgical-site infection after the index orthopedic procedure. The definition used by each study was accepted for the primary analysis. When multiple definitions or time points were reported, independently adjudicated or Centers for Disease Control and Prevention-compatible definitions were preferred, and the longest clinically relevant follow-up within 12 months was selected. For implant-related procedures, infections reported beyond 90 days were retained when the original study considered them related to the index operation.

Secondary infection outcomes included superficial or non-deep surgical-site infection and deep, organ/space, complicated, or implant-associated infection. Safety and stewardship-related outcomes included antibiotic-related complications, acute kidney injury, *C. difficile* infection, antimicrobial resistance, healthcare-associated infection, readmission, reoperation, and mortality. Nonspecific wound findings such as erythema, seroma, drainage, wound dehiscence, or cerebrospinal fluid leakage were not classified as surgical-site infection unless infection was explicitly diagnosed.

Study-specific SSI definitions, surveillance methods, and follow-up periods were retained and were not assumed to be clinically interchangeable across trials. Randomization was considered to support the internal validity of the treatment comparison within each individual trial; however, differences in post-discharge ascertainment and follow-up duration were considered potential sources of indirectness and limited comparability between trials. Studies with shorter or incompletely reported surveillance periods may have failed to capture infections presenting after hospital discharge and may have underestimated delayed implant-associated infections. These differences were considered during interpretation of the pooled results and assessment of certainty of evidence.

### 2.7. Risk-of-Bias Assessment and Certainty of Evidence

Risk of bias in individually randomized trials was assessed using the Cochrane Risk of Bias 2 tool. The cluster-randomized trial was assessed using the corresponding Risk of Bias 2 approach for cluster-randomized designs. Domains included the randomization process, deviations from intended interventions, missing outcome data, measurement of the outcome, and selection of the reported result. Nonrandomized comparative studies included in the qualitative synthesis were assessed using ROBINS-I, considering confounding, participant selection, intervention classification, deviations from intended interventions, missing data, outcome measurement, and selection of the reported result.

Two reviewers independently assessed risk of bias. Disagreements were resolved by discussion or consultation with a third reviewer. Randomized trials were judged as low risk of bias, some concerns, or high risk of bias. Nonrandomized studies were judged as low, moderate, serious, or critical risk of bias, or no information. The certainty of evidence for the main randomized outcomes was evaluated using the GRADE approach, considering risk of bias, inconsistency, indirectness, imprecision, and publication bias. Risk-of-bias visualizations were prepared using the robvis package in R (version 4.5.1).

### 2.8. Statistical Analysis

The primary quantitative synthesis compared shorter versus prolonged systemic prophylaxis for any surgical-site infection using individually randomized trials with extractable treatment-arm data and the published cluster-adjusted effect estimate from the eligible cluster-randomized trial. The shorter prophylaxis group was treated as the experimental group and the prolonged prophylaxis group as the control group. Dichotomous outcomes were summarized using risk ratios with 95% confidence intervals. A risk ratio below 1.00 favoured shorter prophylaxis, whereas a risk ratio above 1.00 favoured prolonged prophylaxis.

Pooled estimates were calculated using a random-effects inverse-variance model on the log risk-ratio scale. A random-effects model was selected a priori because clinical and methodological heterogeneity was expected across surgical procedures, implant use, baseline infection risk, antibiotic agents, prophylaxis-duration contrasts, follow-up periods, and definitions of surgical-site infection. Statistical heterogeneity was interpreted separately from clinical heterogeneity. A low I^2^ value was not considered evidence that the included procedures, populations, or prophylaxis regimens were clinically interchangeable, particularly because the number of trials and infection events was limited. Between-study heterogeneity was assessed using Cochran’s χ^2^ test, I^2^, and τ^2^. A 95% prediction interval was calculated for the primary outcome. Trials with zero events in one treatment arm were included using a continuity correction of 0.5. Studies with insufficient treatment-arm event data were not included in the affected meta-analysis.

Nagata et al. was a cluster-randomized trial in which allocation varied according to hospital and study period. To account for the clustered design, the trial-reported cluster-adjusted risk ratio and 95% confidence interval for surgical-site infection were used in the primary meta-analysis. The adjusted estimate, RR 0.67 (95% CI 0.30–1.49), was entered on the logarithmic scale using the generic inverse-variance method. Published cluster-adjusted estimates were also used for the applicable secondary outcomes. A sensitivity analysis was performed after excluding the cluster-randomized trial entirely to evaluate whether its inclusion influenced the pooled result.

Prespecified subgroup analyses were conducted according to orthopedic procedure type: arthroplasty/reconstruction/implant surgery, spine surgery, closed-fracture fixation, and mixed clean orthopedic surgery. Although procedure-based subgroups were prespecified, these analyses were considered exploratory and hypothesis-generating because each subgroup included few trials and the statistical test for interaction had limited power. The absence of a statistically significant subgroup interaction was not interpreted as evidence that treatment effects were identical across procedure categories. Additional secondary analyses summarized superficial or non-deep infection, deep or complicated infection, antibiotic-related complications, and other infection-related outcomes separately. The outcome-type analysis was interpreted descriptively because the included outcome categories were clinically distinct.

Network meta-analysis was considered but was not performed because the eligible trials did not form a sufficiently connected and clinically coherent network of repeated intervention nodes. Potential duration categories—including single-dose prophylaxis, less than 24 h, 24 h, 48 h, 72 h, continuation until drain removal, and multiday prophylaxis—were generally evaluated in different surgical procedures using different antibiotic agents and in populations with different baseline infection risks and follow-up periods. Each potential intervention node contained few trials and sparse infection events. Consequently, the similarity and transitivity assumptions required for reliable indirect comparisons were not considered sufficiently plausible, and treatment rankings could have been unstable or misleading. The prespecified pairwise comparison of shorter versus prolonged prophylaxis was therefore retained.

Small-study effects were assessed by visual inspection of the funnel plot and Egger’s regression test because at least ten studies contributed to the primary analysis. Sensitivity analyses included exclusion of the cluster-randomized trial and leave-one-out analysis performed by sequentially excluding each randomized trial from the primary meta-analysis. Nonrandomized studies were synthesized narratively and were not pooled with randomized trials. Quantitative analyses were performed using R statistical software (R Foundation for Statistical Computing, Vienna, Austria), with the meta and metafor packages for meta-analysis and sensitivity analyses.

## 3. Results

### 3.1. Study Selection

The database search identified 1035 records, including 601 from PubMed/MEDLINE, 198 from Scopus, and 236 from the Cochrane Central Register of Controlled Trials. After removal of 359 duplicate records and 520 records removed for other reasons, 156 records were screened by title and abstract. Of these, 107 records were excluded, and 49 reports were sought for full-text retrieval. All 49 full-text reports were retrieved and assessed for eligibility.

Thirteen reports were excluded after full-text assessment. Six studies were excluded because they evaluated antibiotic agent selection or dosing rather than prophylaxis duration [3,20,22,23,24,25]. Five studies were excluded because they primarily assessed timing of preoperative prophylaxis without a postoperative duration comparison [4,26,27,28,29]. One placebo-controlled trial was excluded because it evaluated antibiotic prophylaxis versus placebo rather than shorter versus prolonged prophylaxis [21]. One study was excluded because it addressed infection epidemiology in orthopaedic oncology without a comparative duration analysis [30]. Overall, 36 studies were included in the qualitative systematic review, and 11 randomized controlled trials contributed data to the quantitative meta-analysis. The study-selection process is shown in Figure 1.

### 3.2. Characteristics of Included Studies

The 36 studies included in the qualitative synthesis comprised randomized duration-comparison trials, nonrandomized comparative studies, database analyses, historical-control cohorts, and studies addressing duration-related safety or resistance outcomes. The evidence covered several orthopaedic settings, including hip surgery, primary arthroplasty, closed-fracture fixation, spinal surgery, mixed clean orthopaedic procedures, and lower-extremity tumour endoprosthetic reconstruction.

The quantitative synthesis included 11 randomized controlled trials with 3823 analyzed participants. These trials compared single-dose or shorter-duration prophylaxis with multiple-dose or prolonged regimens across hip surgery, instrumented and noninstrumented spine surgery, arthroplasty, closed-fracture fixation, mixed clean orthopaedic surgery, and orthopaedic oncology [5,6,9,10]. Additional randomized evidence included fracture fixation, arthroplasty, tumour endoprosthetic reconstruction, a cluster-randomized clean orthopaedic surgery trial, hip-fracture surgery, and clean orthopaedic trauma surgery [1,2,7,8,11,12,31]. One randomized trial comparing one-day and five-day prophylaxis after lumbar arthrodesis was included in the systematic review but not pooled in the primary SSI meta-analysis because the treatment arm in which the single SSI occurred was not reported separately [32].

The duration contrasts varied across studies. Some trials compared a single preoperative or perioperative dose with multiple-dose regimens, whereas others compared 24-h prophylaxis with 72-h courses, antibiotics continued until drain removal, or multiday prophylaxis. Follow-up periods and SSI definitions also differed, with some trials reporting any SSI and others distinguishing superficial from complicated or deep infections. Detailed information regarding study-specific SSI definitions, post-discharge surveillance methods, maximum follow-up periods, and the time points used in the quantitative synthesis is presented in Supplementary Table S2. Table 1 summarizes the key characteristics of the included studies.

### 3.3. Qualitative Synthesis

The qualitative synthesis included randomized duration-comparison trials and nonrandomized comparative studies that evaluated systemic perioperative antibiotic prophylaxis duration across clean orthopaedic procedures. Overall, the evidence did not show a consistent infection-prevention advantage for prolonged prophylaxis. However, the direction and certainty of findings differed across procedure type, baseline infection risk, study design, and whether prophylaxis was extended only during hospitalization or continued after discharge.

#### 3.3.1. Randomized Duration-Comparison Evidence

The randomized trials formed the most direct evidence base for the review question. These studies evaluated several duration contrasts, including single-dose versus multiple-dose prophylaxis, 24-h versus 72-h prophylaxis, prophylaxis continued until drain removal, and one-day versus multiday regimens [5,6,9,10]. Across hip surgery, spine surgery, arthroplasty, fracture fixation, mixed clean orthopaedic surgery, and orthopaedic oncology, the randomized evidence generally did not demonstrate a consistent reduction in SSI with prolonged postoperative antibiotic administration [1,2,7,8,11]. Several trials had low event rates in both intervention arms, which limited the precision of individual study estimates and made pooled analysis necessary. One additional randomized trial in lumbar arthrodesis was retained in the qualitative synthesis but not included in the primary meta-analysis because the treatment arm in which the single SSI occurred was not clearly reported [32].

#### 3.3.2. Spine Surgery Evidence

Spine surgery represented one of the largest and most clinically heterogeneous evidence areas. Randomized trials in instrumented lumbar fusion, spinal surgery with drains, instrumented spinal fusion, and posterior spinal surgery requiring closed-suction drainage did not show a clear SSI reduction with prolonged prophylaxis compared with shorter regimens [6,7,9,10]. These trials are clinically important because instrumentation and postoperative drains are frequently used as reasons to extend antibiotic prophylaxis. However, the available randomized evidence did not support routine continuation solely on the basis of drain use or instrumentation.

Nonrandomized spine studies showed more variable findings. Some protocol-based or historical comparative studies suggested lower infection rates with longer prophylaxis, particularly in instrumented or higher-risk spinal procedures [33,37,47]. Other studies evaluating postoperative antimicrobial discontinuation, adolescent scoliosis drain-duration protocols, or extended postoperative regimens did not provide uniform evidence that longer prophylaxis consistently improved infection outcomes [34,36,49]. Larger database and cohort analyses also challenged the assumption that postdischarge or prolonged prophylaxis reduces SSI after spinal fusion or lumbar spine surgery [13,17,42]. These inconsistencies likely reflect differences in case complexity, instrumentation, drain protocols, patient comorbidity, local practice patterns, and confounding by indication.

#### 3.3.3. Arthroplasty and Implant-Related Evidence

Arthroplasty and implant-related studies also produced mixed findings. Randomized and comparative evidence in hip surgery and primary arthroplasty generally showed low SSI or PJI event rates, with no consistent signal that multiple-dose or multiday prophylaxis was superior to shorter prophylaxis [5,11,35,38]. The low number of infections in several of these studies limits the ability to exclude small differences between strategies, but the direction of evidence does not clearly favour routine prolongation.

Evidence regarding extended oral antibiotic prophylaxis after total hip and knee arthroplasty was inconsistent. Some high-risk arthroplasty cohorts suggested a lower infection rate after extended oral prophylaxis, particularly in selected patients considered at increased risk of PJI [14,44]. However, more recent comparative studies did not consistently show improved infection-free survivorship or reduced 90-day infection after extended oral prophylaxis in primary total hip or knee arthroplasty [15,16,43]. This inconsistency suggests that any potential benefit of extended oral prophylaxis may be population-specific and difficult to separate from selection bias in nonrandomized designs.

#### 3.3.4. Fracture Fixation, Mixed Orthopaedic Surgery, and Broader Orthopaedic Cohorts

Closed-fracture fixation trials provided direct evidence in orthopaedic trauma performed under clean conditions. These studies compared shorter prophylaxis with additional postoperative or prolonged regimens, but infection events were uncommon and confidence intervals were wide [1,31]. As a result, the fracture-fixation evidence supports inclusion in pooled analysis but does not independently establish a strong advantage for either shorter or prolonged prophylaxis.

Mixed clean orthopaedic evidence broadened the clinical scope of the review. The cluster-randomized trial in clean orthopaedic surgery compared discontinuation within 24 h with continuation for 24–48 h and contributed important pragmatic evidence across routine orthopaedic procedures [8]. Additional cohort evidence from broad orthopaedic populations suggested that prophylaxis duration remains variable in real-world practice and that duration-related findings may differ according to procedure subgroup, trauma status, and institutional protocol [41,45,46]. These studies support the clinical relevance of the review question beyond a single procedure type, but their heterogeneity limits direct causal interpretation.

#### 3.3.5. Orthopaedic Oncology Evidence

Orthopaedic oncology was represented in the randomized evidence by lower-extremity bone-tumour resection followed by endoprosthetic reconstruction. This population differs from routine clean orthopaedic surgery because procedures are often longer, implants are larger, soft-tissue dissection is more extensive, and baseline infection risk is higher. The oncology trial was therefore clinically important but not directly interchangeable with routine arthroplasty, fracture fixation, or standard spine surgery [2]. Its inclusion strengthened the breadth of the evidence base, while also reinforcing the need to interpret procedure-specific subgroup findings carefully.

#### 3.3.6. Safety, Resistance, and Stewardship-Related Evidence

Safety and stewardship outcomes were reported less consistently than SSI, but the available evidence raised important concerns about prolonged antibiotic exposure. Longer prophylaxis duration was associated with postoperative adverse outcomes in large cohort evidence, including outcomes relevant to antimicrobial stewardship such as acute kidney injury and *Clostridioides difficile* infection [17,18]. Resistance-focused arthroplasty evidence also suggested that extended oral antibiotic prophylaxis may influence antimicrobial-resistance patterns among subsequent periprosthetic joint infections [19]. Practice-based orthopaedic implant data further showed variability in antimicrobial selection, duration, adherence to recommendations, adverse reactions, and costs [46].

Overall, the qualitative synthesis supports the main quantitative finding: prolonged systemic prophylaxis has not shown a consistent SSI-prevention advantage across clean orthopaedic surgery. The strongest direct evidence comes from randomized duration-comparison trials, while nonrandomized studies provide useful context regarding real-world prescribing, high-risk populations, safety, resistance, and implementation. Taken together, the evidence suggests that routine prolongation should not be assumed beneficial across all clean orthopaedic procedures and should be balanced against antimicrobial harms and stewardship priorities.

### 3.4. Quantitative Synthesis

#### 3.4.1. Primary Outcome: Any Surgical-Site Infection

**Figure 2:**
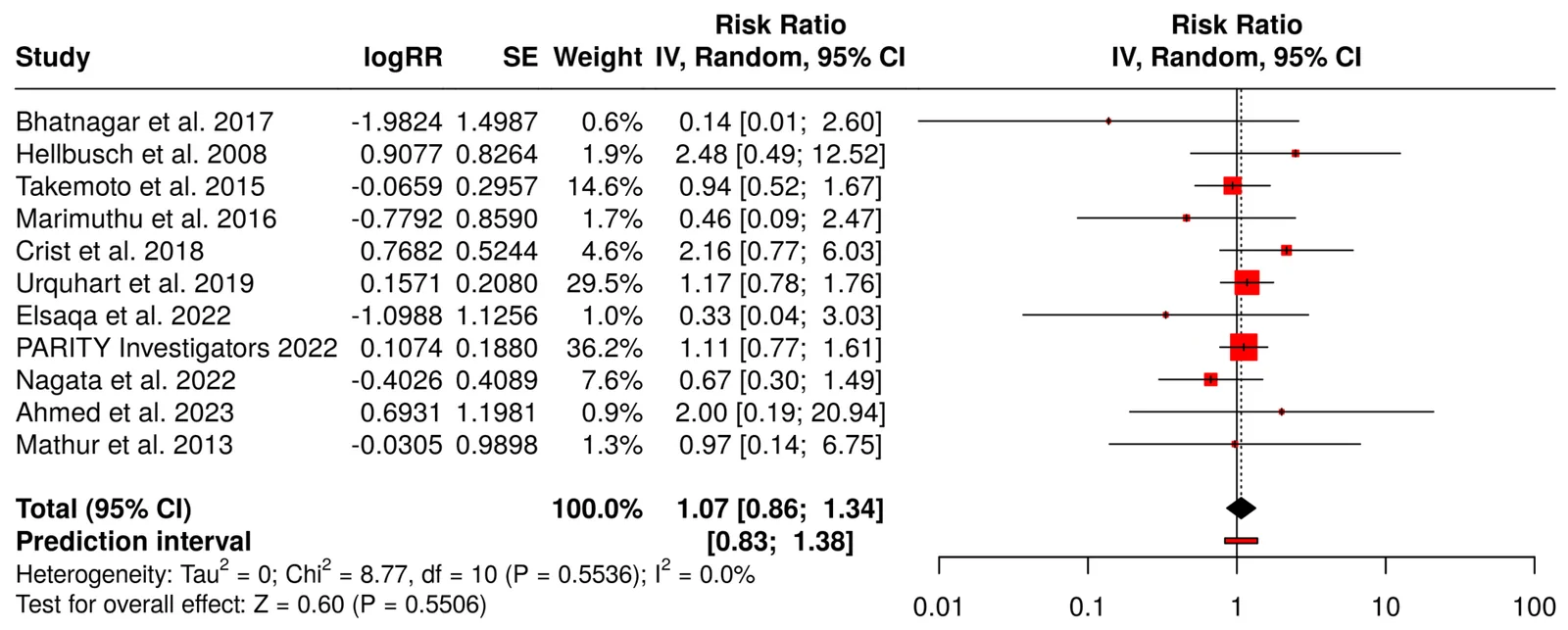
Forest plot comparing shorter versus prolonged systemic perioperative antibiotic prophylaxis for any surgical-site infection. The cluster-adjusted estimate reported by Nagata et al. was used for the cluster-randomized trial [1,2,5,6,7,8,9,10,11,12,31].

Eleven randomized trials involving 3823 participants were included in the primary meta-analysis of any SSI [1,2,5,6,7,8,9,10,11,12,31]. After incorporation of the trial-reported cluster-adjusted estimate for the cluster-randomized study, the pooled random-effects risk ratio was 1.07 (95% CI 0.86–1.34). The confidence interval crossed the line of no effect and remained compatible with both a possible reduction and a possible increase in SSI. The prediction interval was 0.83–1.38. Observed statistical heterogeneity was negligible (I^2^ = 0.0%; τ^2^ = 0; χ^2^ = 8.77, df = 10, *p* = 0.554), and the overall test for effect was not statistically significant (Z = 0.60, *p* = 0.551). However, the low statistical heterogeneity should not be interpreted as an absence of clinical heterogeneity across the included procedures, prophylaxis regimens, follow-up periods, and SSI definitions. The primary forest plot is shown in Figure 2.

#### 3.4.2. Publication Bias and Small-Study Effects

**Figure 3:**
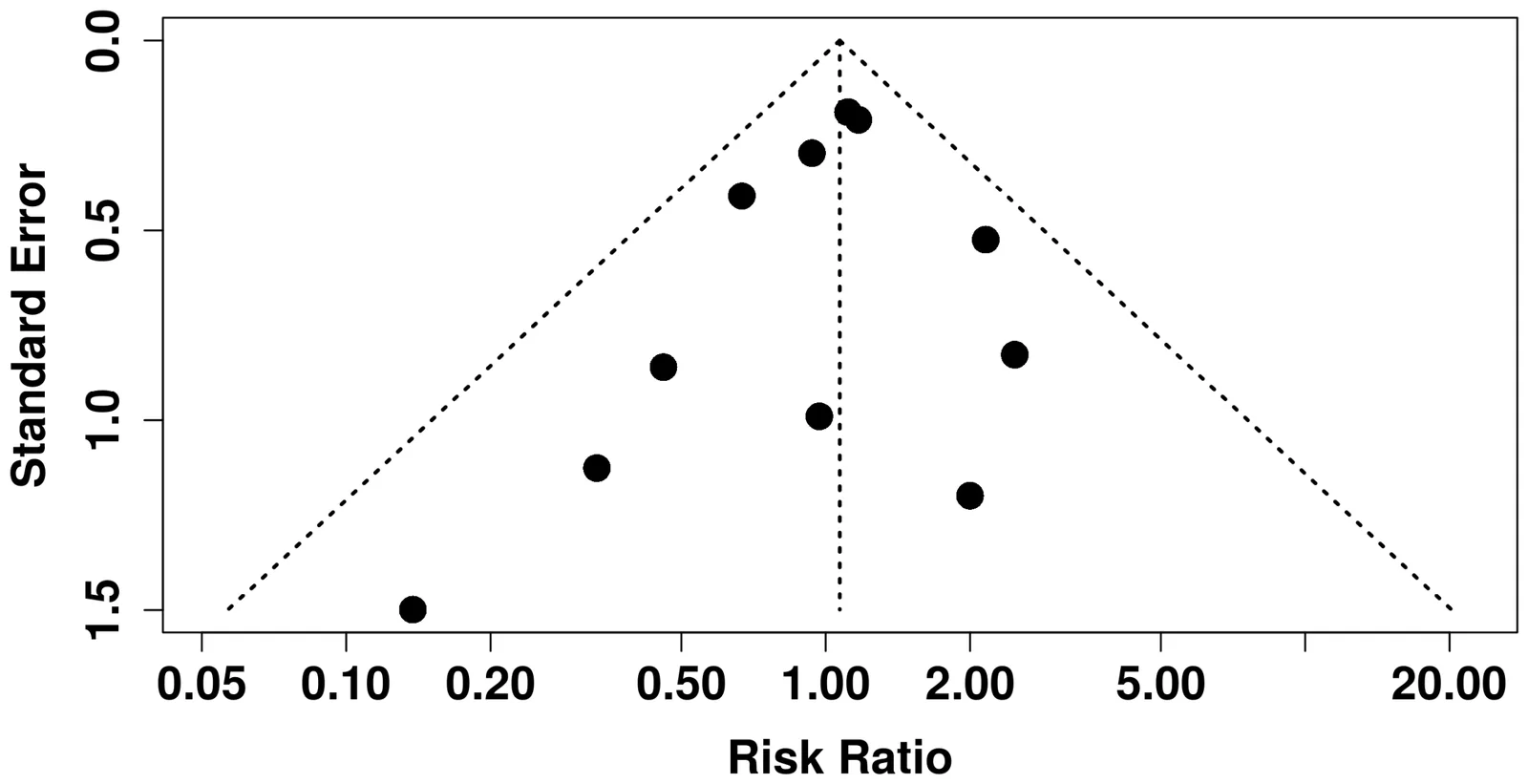
Funnel plot assessing small-study effects for the primary surgical-site infection analysis. The vertical dotted line represents the pooled risk ratio, and the diagonal dotted lines represent pseudo-95% confidence limits.

Visual inspection of the funnel plot did not suggest marked asymmetry. Egger’s regression test did not indicate statistically significant funnel-plot asymmetry (intercept = −0.41, t = −0.84, *p* = 0.422). However, the assessment had limited power because only 11 trials contributed and several trials reported sparse infection events. The funnel plot is shown in Figure 3.

#### 3.4.3. Subgroup Analysis by Orthopaedic Procedure

Exploratory subgroup analysis was performed according to orthopaedic procedure type. In the arthroplasty/reconstruction/implant surgery subgroup, the pooled estimate was RR 0.70 (95% CI 0.23–2.08; I^2^ = 32.7%). In the spine surgery subgroup, the pooled estimate was RR 1.09 (95% CI 0.79–1.50; I^2^ = 0%). The closed-fracture fixation subgroup produced a pooled estimate of RR 1.83 (95% CI 0.79–4.28; I^2^ = 0%), with considerable imprecision. The mixed clean orthopaedic surgery subgroup consisted of one cluster-randomized trial and produced a cluster-adjusted estimate of RR 0.67 (95% CI 0.30–1.49).

**Figure 4:**
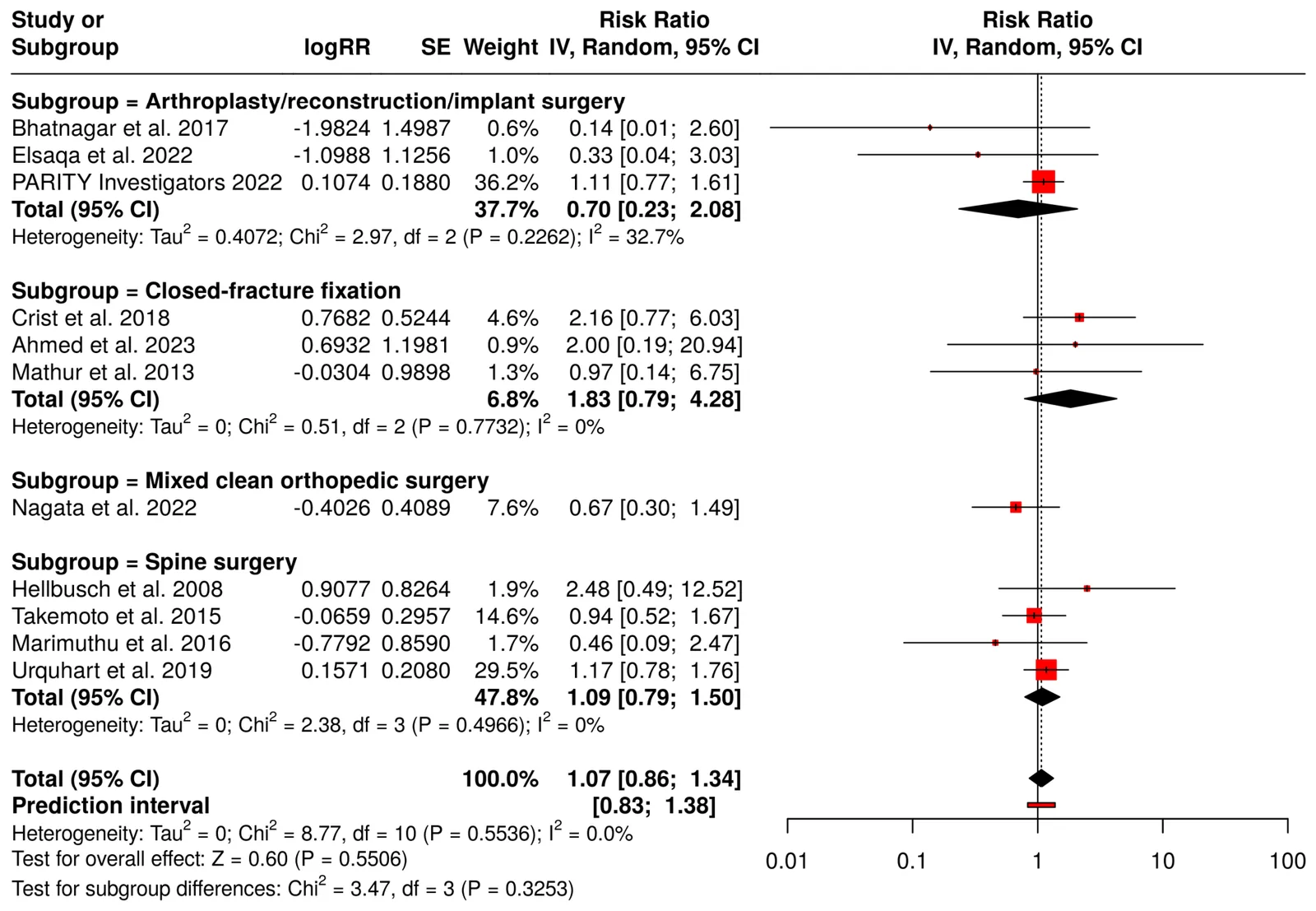
Exploratory subgroup forest plot comparing shorter versus prolonged systemic perioperative antibiotic prophylaxis according to orthopaedic procedure type. The cluster-adjusted estimate reported by Nagata et al. was used for the cluster-randomized trial [1,2,5,6,7,8,9,10,11,12,31].

Across all procedure categories, the pooled estimate was RR 1.07 (95% CI 0.86–1.34). The test for subgroup differences was not statistically significant (χ^2^ = 3.47, df = 3, *p* = 0.325). These findings should be interpreted as exploratory and hypothesis-generating because each subgroup contained only one to four trials and the interaction test had limited statistical power. The absence of a statistically significant interaction does not establish identical treatment effects across procedure categories. The subgroup forest plot is shown in Figure 4.

#### 3.4.4. Secondary Infection-Related and Safety Outcomes

Secondary analyses were performed according to infection depth and selected safety or infection-related outcomes. For superficial or non-deep SSI, seven trials contributed data, and the pooled estimate showed no statistically significant difference between shorter and prolonged prophylaxis (RR = 1.19, 95% CI 0.79–1.77; I^2^ = 0%). For deep or complicated SSI, seven trials contributed estimable effects, while one additional double-zero trial contributed no weight to the risk-ratio analysis. After incorporation of the cluster-adjusted estimate from Nagata et al., the pooled result was RR 0.85 (95% CI 0.55–1.32; I^2^ = 0%).

Antibiotic-related complications were reported in one randomized trial and were less frequent with shorter prophylaxis (RR = 0.31, 95% CI 0.12–0.85). *Clostridioides difficile*–associated colitis was examined separately. In the PARITY trial, colitis occurred in 4 of 311 participants receiving one-day prophylaxis and 11 of 293 receiving five-day prophylaxis, corresponding to RR 0.34 (95% CI 0.11–1.06) for shorter versus prolonged prophylaxis [2]. Nagata et al. reported no pseudomembranous-colitis events in either treatment group (0/633 versus 0/578) [8]. Because only one trial provided an estimable risk ratio, no multi-study pooled estimate was calculated for this outcome.

**Figure 5:**
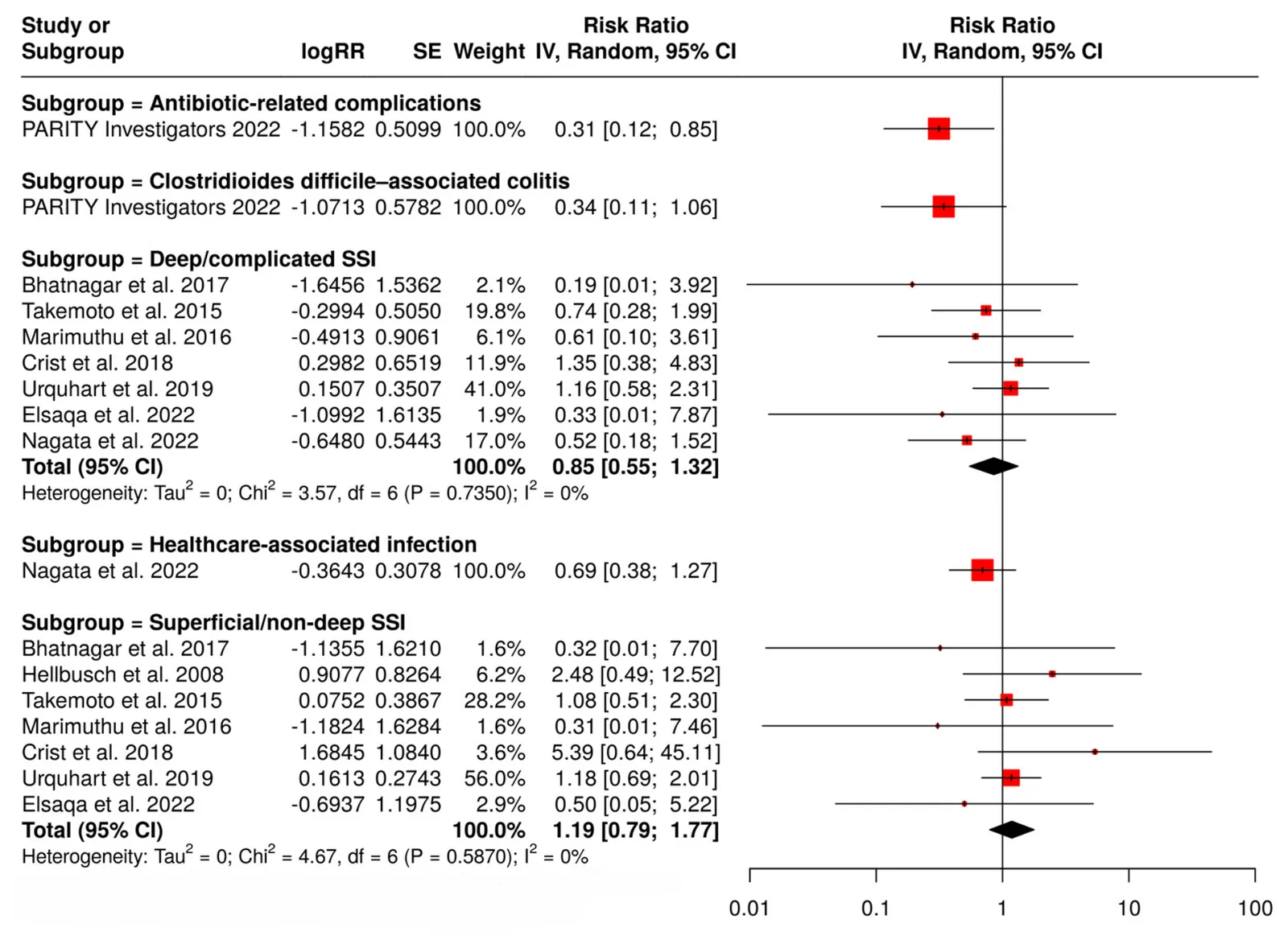
Descriptive forest plot of secondary infection-related and safety outcomes according to outcome type. Outcome categories, including antibiotic-related complications and Clostridioides difficile–associated colitis, were analyzed separately because they represent clinically distinct endpoints and were not combined into a single overall estimate. Cluster-adjusted estimates were used for applicable outcomes reported by Nagata et al.; its double-zero C. difficile outcome was non-estimable and is described in the text. The displayed point estimate for healthcare-associated infection was reconstructed from the published rounded confidence limits and therefore differs slightly from the source-reported adjusted RR of 0.70 because of rounding [1,2,5,6,7,8,9,10,11].

Healthcare-associated infection was reported in one cluster-randomized trial, with no statistically significant difference between groups using the cluster-adjusted estimate (RR = 0.70, 95% CI 0.38–1.27). Because these outcomes represent clinically distinct endpoints, they were analyzed and displayed separately and were not combined into a single overall secondary-outcome estimate (Figure 5).

### 3.5. Sensitivity Analysis

A dedicated sensitivity analysis was performed to evaluate the influence of the cluster-randomized trial. After excluding Nagata et al., the remaining 10 individually randomized trials included 2612 participants. The pooled estimate remained non-significant (RR = 1.11, 95% CI 0.88–1.40), with no observed statistical heterogeneity (I^2^ = 0.0%; τ^2^ = 0; χ^2^ = 7.34, df = 9, *p* = 0.601). This was consistent with the primary analysis incorporating the cluster-adjusted estimate.

Leave-one-out sensitivity analysis was subsequently performed by excluding each trial in turn. Pooled estimates ranged from 1.03 to 1.11, all confidence intervals crossed the line of no effect, and I^2^ remained 0% throughout. These findings indicate that the overall interpretation was not dependent on any single trial. The leave-one-out sensitivity-analysis results are summarized in Table 2.

### 3.6. Risk-of-Bias Assessment

**Figure 6:**
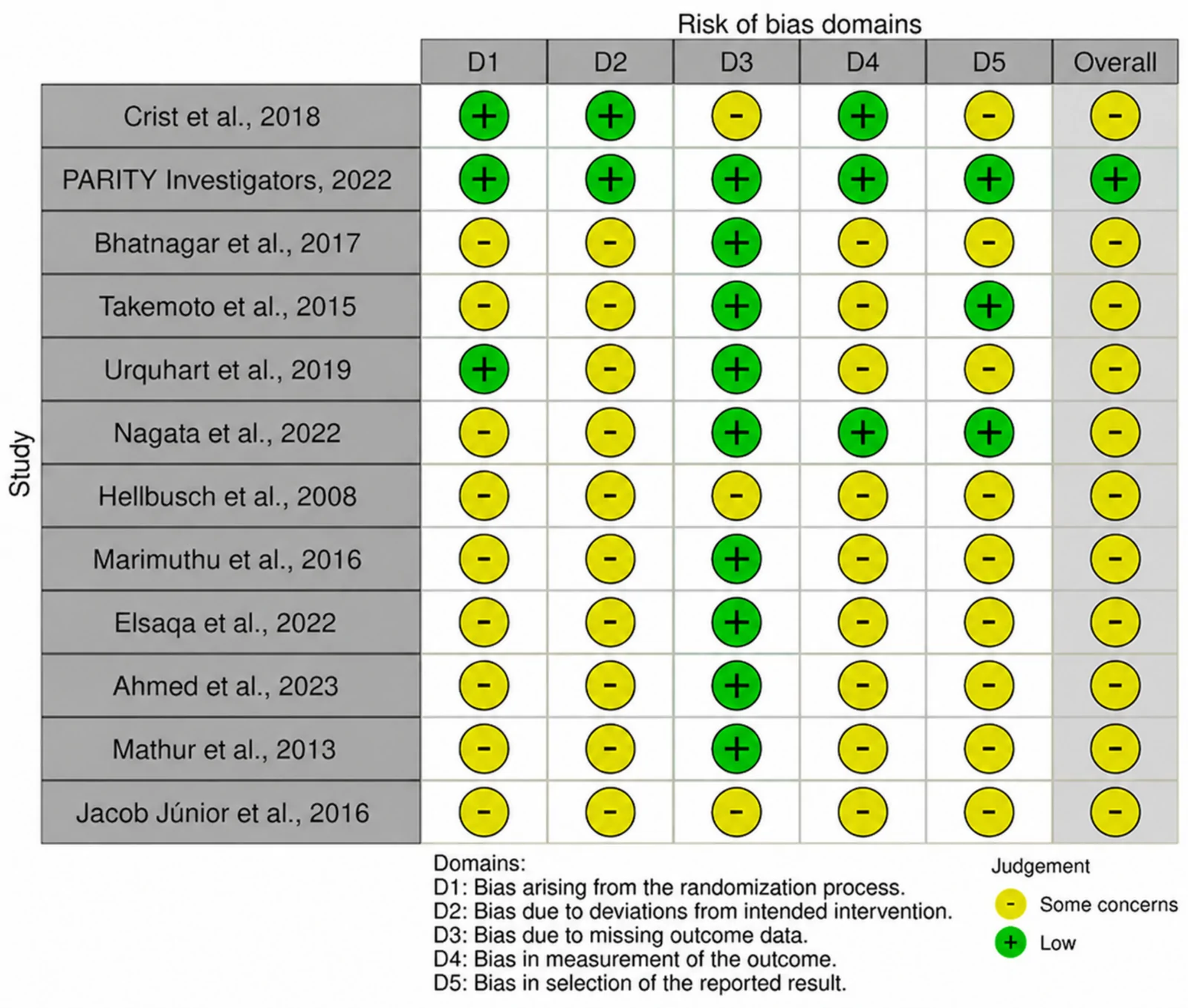
Risk-of-bias assessment of randomized trials using the RoB 2 framework. Judgments are shown across the five RoB 2 domains and the overall judgment for each randomized trial [1,2,5,6,7,8,9,10,11,12,31,32].

Risk-of-bias assessment for randomized trials was performed using the RoB 2 framework and is presented in Figure 6. Overall, most randomized trials were judged as either low risk or having some concerns. The main concerns were related to limited reporting of allocation procedures, open-label perioperative management, post-randomization exclusions, sparse event counts, and incomplete reporting of treatment-arm outcome data. The cluster-randomized trial was assessed within the RoB 2 framework, with its cluster design considered during interpretation.

**Figure 7:**
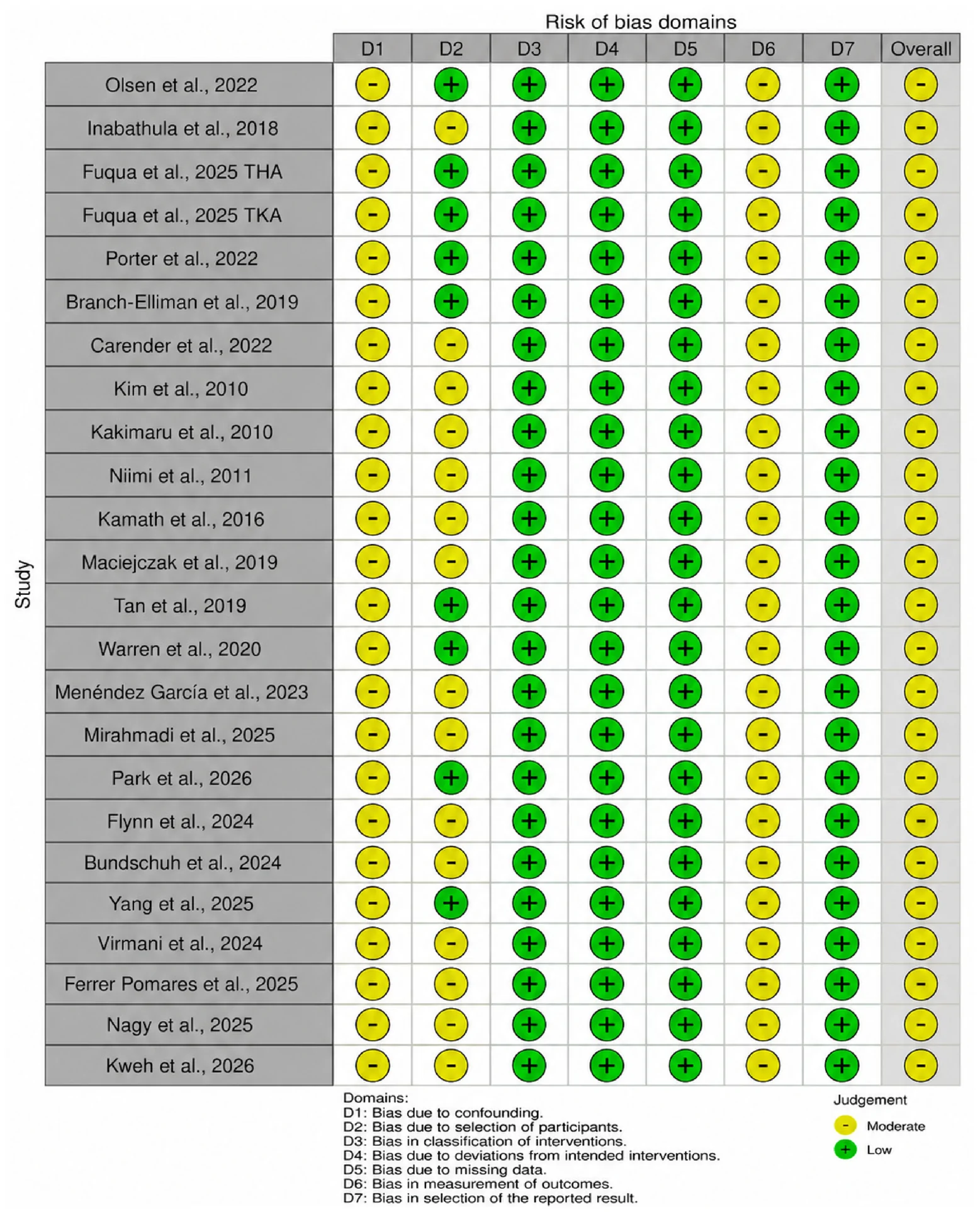
Risk-of-bias assessment of nonrandomized comparative studies using ROBINS-I. Judgments are shown across the seven ROBINS-I domains and the overall judgment for each nonrandomized study [13,14,15,16,17,18,19,33,34,35,36,37,38,39,40,41,42,43,44,45,46,47,48,49].

Risk-of-bias assessment for nonrandomized comparative studies was performed using ROBINS-I and is presented in Figure 7. Most nonrandomized studies were judged as having low to moderate risk of bias. The most common concerns were confounding by indication, retrospective exposure assignment, historical-control designs, surgeon- or protocol-driven antibiotic selection, and incomplete adjustment for patient-level infection risk. These findings support the decision to pool only randomized trials in the primary meta-analysis and to synthesize nonrandomized evidence qualitatively.

### 3.7. Certainty of Evidence

The certainty of evidence was assessed using the GRADE approach and is summarized in Table 3. The certainty of evidence for the primary SSI outcome was rated as low because of risk-of-bias concerns and imprecision related to sparse infection events. Certainty was also low for superficial/non-deep SSI, deep/complicated SSI, antibiotic-related complications, and healthcare-associated infection because these outcomes were limited by small numbers of events, procedure heterogeneity, and, for some outcomes, reliance on a single trial.

## 4. Discussion

This systematic review and meta-analysis found no statistically significant difference in the risk of any surgical-site infection (SSI) between shorter-duration and prolonged systemic perioperative antibiotic prophylaxis in clean orthopaedic surgery. Across 11 randomized trials involving 3823 participants, the pooled estimate was close to the line of no effect, with negligible statistical heterogeneity. Similar findings were observed across procedure-based subgroup analyses and in the separate analyses of superficial and deep or complicated SSI. These findings suggest that, within the limitations of the available randomized evidence, routine prolongation of systemic antibiotic prophylaxis does not provide a clearly demonstrable reduction in SSI compared with shorter regimens. Importantly, this should not be interpreted as proof of equivalence between strategies, but rather as an absence of statistically detectable benefit from prolonged prophylaxis in the currently available trial evidence.

The pooled estimate should be interpreted as an average effect across clinically diverse settings rather than as evidence of a uniform treatment effect throughout clean orthopaedic surgery. The randomized trials differed substantially in surgical procedure, implant burden, baseline infection risk, antibiotic agent, dosing schedule, duration contrast, drain use, follow-up period, and SSI definition [1,2,5,6,7,8,9,10,11,12,31]. Routine arthroplasty and hip surgery [5,11], closed-fracture fixation [1,31], instrumented spine surgery with postoperative drains [6,7,9,10], mixed clean orthopaedic surgery [8], and tumour endoprosthetic reconstruction [2] are not clinically interchangeable. Although observed statistical heterogeneity was low, this does not eliminate the substantial clinical heterogeneity or exclude procedure-specific differences, particularly because the small number of trials and sparse events limited the power to detect between-study variation. The pooled result should therefore not be generalized as demonstrating that shorter prophylaxis is sufficient for every clean orthopaedic procedure.

The orthopaedic oncology evidence requires a separate clinical interpretation. Tumour endoprosthetic reconstruction differs substantially from routine clean orthopaedic surgery because of larger implants, longer operative times, extensive soft-tissue dissection, and higher baseline infection risk. In the PARITY trial, one-day and five-day prophylaxis produced similar infection outcomes, while antibiotic-related complications were more frequent in the prolonged group [2]. This result is important because it suggests that even in a high-risk reconstructive setting, longer prophylaxis may not translate into a clear infection-prevention benefit. However, orthopaedic oncology should not be used as a simple proxy for routine arthroplasty, fracture fixation, or standard spine surgery. The microbiology and timing of infections after endoprosthetic reconstruction may also differ from other clean orthopaedic procedures, as shown by cohort evidence examining endoprosthetic infections in this population [30].

Important uncertainty remains in higher-risk procedures. Orthopaedic oncology evidence was driven principally by a single randomized trial of lower-extremity tumour resection and endoprosthetic reconstruction, and its findings should not be assumed to apply to all oncologic reconstructions [2]. Similarly, evidence relevant to complex instrumented spine surgery was derived from a small number of randomized trials with heterogeneous populations, instrumentation, drain protocols, antibiotic regimens, duration contrasts, and follow-up periods [6,7,9,10]. These data are insufficient to establish procedure-specific equivalence between shorter and prolonged prophylaxis. Adequately powered randomized trials are required in orthopaedic oncology, revision or multilevel instrumented spine surgery, and other complex implant-related procedures, with stratification according to host risk, instrumentation, revision status, drain use, and operative complexity.

The qualitative synthesis showed that nonrandomized studies do not provide a consistent counterargument to the randomized findings. Some cohort and before-and-after studies suggested lower SSI rates after extended oral or prolonged prophylaxis protocols, particularly in selected high-risk arthroplasty or instrumented spine populations [14,40,44,47]. However, other large database and institutional studies did not find a reduction in SSI or periprosthetic joint infection with postdischarge or extended antibiotic prophylaxis [13,15,16,43]. This inconsistency is unsurprising because nonrandomized studies are strongly affected by patient selection, surgeon preference, local protocols, changes in infection-control practice, and confounding by indication. Patients receiving extended antibiotics are often those considered to be at higher risk, while before-and-after designs may be influenced by broader temporal improvements in surgical care.

The distinction between randomized and nonrandomized evidence is therefore essential. Observational studies are valuable for describing real-world prescribing patterns, high-risk subgroups, and uncommon harms, but they are less reliable for estimating the causal effect of prophylaxis duration. For example, studies of postdischarge antibiotics after spinal fusion and large cohort analyses of lumbar spine surgery provide useful information about practice and outcomes, but they cannot fully eliminate confounding from procedural complexity or risk-based prescribing [17,39,42]. Similarly, extended oral antibiotic prophylaxis after arthroplasty remains controversial because positive findings from high-risk cohorts have not been consistently reproduced in later comparative studies [14,16,43]. In this context, the randomized trial evidence should remain the primary basis for conclusions about comparative effectiveness.

Continued use of prolonged prophylaxis likely reflects concern about the severe consequences of implant-associated infection, perceived host or procedural risk, the presence of drains or spinal instrumentation, surgeon-specific preference, and persistence of historical institutional protocols [6,7,9,10,39,41,46,48,49]. Risk aversion is understandable in complex reconstruction, but perceived risk does not establish that prolonged prophylaxis is effective. Variation may also arise when protocolized prophylaxis is not clearly distinguished from empiric treatment initiated for postoperative fever, wound drainage, erythema, or another concern for infection. Stewardship interventions should therefore emphasize procedure-specific protocols, documentation of indication and intended stop time, and review of deviations from standard duration.

The results also reinforce that antibiotic duration should not be considered in isolation from the broader quality of prophylaxis. Appropriate agent selection, dose, timing, and redosing are necessary before duration can be meaningfully evaluated. Arthroplasty studies have shown that alternative antibiotics to cefazolin, vancomycin dosing problems, and dual prophylaxis strategies may influence infection risk and adverse events [3,22,23,24,25]. Timing studies similarly demonstrate that delayed or poorly timed preoperative prophylaxis is a modifiable risk factor for SSI and that improving timing practices can enhance prophylaxis quality [4,26,27,28,29]. Therefore, a short prophylaxis regimen should not mean minimal or careless prophylaxis; rather, it should mean an optimized perioperative regimen that is appropriately selected, correctly dosed, administered on time, and discontinued when further benefit is unlikely.

The stewardship implications are important. Prolonged prophylaxis exposes patients to additional antibiotics without clear evidence of lower SSI in the pooled randomized analysis. This additional exposure is not harmless. Large surgical cohort evidence has associated longer prophylaxis duration with antimicrobial-associated adverse events, including acute kidney injury and *Clostridioides difficile* infection [18]. Orthopaedic-specific studies also raise concerns regarding nephrotoxicity with broader regimens and resistance patterns among subsequent periprosthetic infections after extended oral prophylaxis [19,23]. Practice-based studies further show that real-world antimicrobial prophylaxis in orthopaedic implant surgery can be heterogeneous and costly [46]. Taken together, these findings support antimicrobial-stewardship strategies that discourage routine postoperative continuation unless a clear patient- or procedure-specific rationale exists.

The review has several strengths. First, the primary quantitative synthesis was restricted to randomized duration-comparison trials, reducing the risk that the main pooled estimate would be driven by confounded observational evidence. Second, nonrandomized studies were not discarded but were used to interpret consistency, external validity, safety, and implementation. Third, the review separated overall SSI from superficial, deep or complicated infection, and safety-related outcomes. Fourth, sensitivity analyses indicated that the overall pooled estimate was not dependent on a single trial, while exploratory subgroup analyses were used to examine—but not confirm—possible procedure-specific differences. Finally, the review addressed not only infection prevention but also antimicrobial harms and stewardship, which are central to the clinical decision about prophylaxis duration.

Several limitations should be acknowledged. Many included trials were small, and SSI events were uncommon, resulting in wide confidence intervals in individual studies. Some trials had methodological concerns, including unclear randomization procedures, open-label designs, post-randomization exclusions, or incomplete reporting of outcomes. The included procedures were clinically heterogeneous, ranging from routine arthroplasty and closed-fracture fixation to spinal instrumentation and tumour endoprosthetic reconstruction. Antibiotic agents, dosing schedules, follow-up durations, and SSI definitions also varied across studies. Outcome ascertainment also differed across trials. Some studies included structured surveillance after hospital discharge, whereas others relied primarily on inpatient records or incompletely described follow-up. Shorter surveillance periods may have failed to capture infections presenting after discharge and may particularly have underestimated delayed implant-associated infections. Although randomization supports valid treatment comparisons within individual trials, variation in SSI definitions, surveillance methods, and follow-up duration limits direct comparability between studies. The pooled estimate should therefore be interpreted as a synthesis of study-defined SSI outcomes rather than a completely harmonized endpoint. Some nonrandomized reports also did not clearly distinguish protocolized postoperative prophylaxis from antibiotics initiated because of fever, wound drainage, erythema, positive cultures, or another clinical concern for infection. This may have introduced exposure misclassification and confounding by indication because such prescribing represents empiric or definitive treatment rather than routine prophylaxis. One randomized trial comparing one-day with five-day prophylaxis after lumbar arthrodesis could not be pooled for the primary SSI outcome because the treatment arm containing the infection event was not reported [32]. In addition, the outcome-type analysis included different infection depths and safety outcomes, which should be interpreted descriptively rather than as interchangeable endpoints.

Most randomized trials reported few SSI events, and several individual estimates had wide confidence intervals [1,2,5,6,7,8,9,10,11,12,31]. Consequently, the meta-analysis had limited statistical power to exclude modest but clinically important benefit or harm. The absence of a statistically significant difference should not be interpreted as evidence that shorter and prolonged prophylaxis are equivalent. This review was not designed as a noninferiority analysis, and no prespecified clinical noninferiority margin was applied across the pooled trials. The findings indicate that prolonged prophylaxis has not demonstrated a consistent additional SSI-prevention benefit in the available randomized evidence [1,2,5,6,7,8,9,10,11,12,31]; they do not establish that shorter prophylaxis is equally effective in every patient or procedure.

Although visual inspection of the funnel plot and Egger’s regression test did not indicate statistically significant asymmetry, the assessment had limited power because only 11 trials contributed and several trials reported sparse infection events. Publication-bias assessment should therefore be interpreted cautiously.

Future research should focus on adequately powered, multicentre randomized trials using standardized SSI definitions, procedure-specific stratification, and consistent reporting of superficial and deep infections. Trials should report antibiotic-related complications, acute kidney injury, *C. difficile* infection, antimicrobial resistance, readmission, reoperation, and cost outcomes in addition to SSI. Longer follow-up may be required for implant-associated infections, but early and late infections should be reported separately. Future studies should also distinguish routine clean orthopaedic procedures from genuinely high-risk subgroups rather than applying extended prophylaxis broadly. Clear reporting of randomization, allocation concealment, adherence to assigned prophylaxis, and reasons for postoperative antibiotic continuation will improve the interpretability of future evidence.

In clinical practice, the available randomized evidence does not support routine systemic prophylaxis beyond 24 h after clean orthopaedic surgery [1,2,5,6,7,8,9,10,11,12,31]. The presence of a postoperative drain or spinal instrumentation alone should not justify continued prophylaxis because the available randomized spine trials did not demonstrate a consistent additional SSI-prevention benefit from prolonged administration [6,7,9,10]. Evidence remains limited in orthopaedic oncology, revision surgery, and complex multilevel instrumented spine procedures; however, this uncertainty should not be interpreted as evidence supporting automatic extension. Where prolonged administration is considered, the indication, intended duration, and planned review or stop date should be clearly documented. Antibiotics initiated because of suspected or confirmed postoperative infection should be classified and managed as treatment rather than prophylaxis. These decisions should also consider the additional risks associated with prolonged exposure, including acute kidney injury, Clostridioides difficile infection, antimicrobial resistance, and increased cost [17,18,19,46].

## 5. Conclusions

Prolonged systemic perioperative antibiotic prophylaxis did not demonstrate a consistent additional reduction in surgical-site infection compared with shorter regimens. Routine prophylaxis beyond 24 h should therefore be avoided after clean orthopaedic surgery in the absence of suspected or confirmed infection. Implant placement, spinal instrumentation, or the presence of a drain alone does not justify automatic extension. Sparse events, heterogeneous procedures, variable SSI definitions and follow-up periods, and limited evidence in higher-risk settings prevent conclusions of universal equivalence between regimens. In orthopaedic oncology and complex instrumented spine surgery, uncertainty should prompt adequately powered trials and carefully documented multidisciplinary decisions rather than routine prolonged prescribing. Antibiotics administered for suspected or confirmed postoperative infection should be classified and managed as treatment rather than prophylaxis.

## Figures and Tables

**Table 1:** Characteristics of studies included in the systematic review.

Study	Design	Procedure/Population	Sample Size	Prophylaxis Comparison or Exposure	Main Outcome/Follow-Up	Role in Review
Bhatnagar et al., 2017 [5]	Prospective randomized trial	Hip joint surgery with implant use	104	Single preoperative cefazolin dose vs. multiple injectable antibiotic doses	SSI; 0/53 vs. 3/51 infections	Included in primary meta-analysis
Hellbusch et al., 2008 [9]	Prospective randomized comparative trial	Instrumented lumbar fusion	233 analyzed; 269 randomized	Single-dose prophylaxis vs. extended multiple-dose regimen including postoperative oral antibiotics	SSI; 5/117 vs. 2/116 infections	Included in primary meta-analysis
Kim et al., 2010 [33]	Prospective nonrandomized surgeon-protocol cohort	Spinal surgery	548	48-h vs. 72-h antimicrobial prophylaxis	SSI after spinal surgery	Qualitative synthesis only
Takemoto et al., 2015 [6]	Prospective randomized trial	Spinal surgery with closed-suction drain	314	24-h prophylaxis vs. prophylaxis continued for drain duration	SSI; 21/170 vs. 19/144 infections	Included in primary meta-analysis
Marimuthu et al., 2016 [10]	Prospective randomized comparative trial	Instrumented spinal fusion	326	24-h vs. 72-h antimicrobial prophylaxis	SSI; 2/170 vs. 4/156 infections	Included in primary meta-analysis
Crist et al., 2018 [1]	Randomized double-blind placebo-controlled trial	Closed-fracture ORIF	160 analyzed; 227 randomized	Pre/intraoperative cefazolin plus placebo vs. additional 23-h postoperative cefazolin	SSI to fracture union; 10/77 vs. 5/83 infections	Included in primary meta-analysis
Urquhart et al., 2019 [7]	Randomized controlled trial	Posterior spinal surgery requiring drain	552	24-h vs. 72-h postoperative antibiotics	Complicated and superficial SSI; derived any SSI 44/282 vs. 36/270	Included in primary meta-analysis
Elsaqa et al., 2022 [11]	Prospective randomized controlled trial	Primary joint arthroplasty	60	One-day vs. three-day antibiotic prophylaxis	SSI; 1/30 vs. 3/30 infections	Included in primary meta-analysis
PARITY Investigators, 2022 [2]	Multicentre randomized clinical trial	Lower-extremity bone tumour resection with endoprosthetic reconstruction	604	One-day vs. five-day postoperative intravenous prophylaxis	SSI and antibiotic-related complications; 52/311 vs. 44/293 infections	Included in primary meta-analysis
Nagata et al., 2022 [8]	Cluster randomized noninferiority trial	Clean orthopaedic surgery	1211	Prophylaxis discontinued within 24 h vs. 24–48 h	Healthcare-associated infection and SSI; 14/633 vs. 19/578 SSI	Included in primary meta-analysis
Ahmed et al., 2023 [12]	Randomized prospective pilot study	Hip-fracture surgery	62	Single-dose prophylaxis vs. three-dose prophylaxis	SSI; 2/31 vs. 1/31 infections	Included in primary meta-analysis
Mathur et al., 2013 [31]	Block-randomized controlled trial	Clean orthopaedic surgery/closed-fracture fixation	197	Short-course cefuroxime vs. prolonged broader-spectrum prophylaxis	SSI; 2/100 vs. 2/97 infections	Included in primary meta-analysis
Jacob Júnior et al., 2016 [32]	Prospective randomized trial	Lumbar arthrodesis	43	One-day vs. five-day antibiotic prophylaxis	Wound complications; one SSI reported without treatment-arm attribution	Qualitative synthesis only
Kakimaru et al., 2010 [34]	Prospective historical before-and-after cohort	Spinal decompression surgery	284	Postoperative antimicrobial prophylaxis vs. no postoperative antimicrobial prophylaxis	SSI after decompression surgery	Qualitative synthesis only
Niimi et al., 2011 [35]	Retrospective historical-control comparative cohort	Arthroplasty	327	One-day antibiotic infusion vs. three or more days	Postoperative infection; no infections reported	Qualitative synthesis only
Kamath et al., 2016 [36]	Retrospective comparative cohort	Posterior spinal fusion for adolescent idiopathic scoliosis	226	Two postoperative doses vs. antibiotics until drain removal	SSI after posterior fusion	Qualitative synthesis only
Maciejczak et al., 2019 [37]	Prospective nonrandomized before-and-after cohort	Spine surgery; instrumented and noninstrumented	5208	Single-dose vs. 72-h prophylaxis protocol	SSI; instrumented-spine SSI 5.3% vs. 2.2%	Qualitative synthesis only
Tan et al., 2019 [38]	Large retrospective cohort	Total joint arthroplasty	20,682	Single-dose vs. multiple-dose perioperative prophylaxis	PJI/SSI after arthroplasty	Qualitative synthesis only
Warren et al., 2020 [39]	Multicentre retrospective cohort	Spinal fusion	8652	Postdischarge prophylactic antibiotic use vs. no postdischarge prophylaxis	Frequency and predictors of postdischarge antibiotics	Qualitative synthesis only
Olsen et al., 2022 [13]	Large retrospective commercial-claims cohort	Spinal fusion	156,446	Postdischarge prophylactic antibiotics vs. none	SSI after spinal fusion	Qualitative synthesis only
Porter et al., 2022 [17]	Retrospective national Veterans Health Administration cohort	Lumbar spine surgery	6198	Increasing duration of antimicrobial prophylaxis	SSI, AKI, C. difficile infection, LOS, reoperation	Qualitative synthesis only
Menéndez García et al., 2023 [40]	Retrospective cohort	Instrumented spinal fusion	901	Standard prophylaxis vs. extended oral prophylaxis until suture removal	SSI after instrumented fusion	Qualitative synthesis only
Mirahmadi et al., 2025 [41]	Retrospective comparative cohort	Mixed orthopaedic surgery including TKA, THA, ACL reconstruction, and hip fixation	398	Short-term prophylaxis < 24 h vs. extended oral antibiotics	SSI and postoperative complications at three months	Qualitative synthesis only
Park et al., 2026 [42]	Nationwide retrospective cohort	Spine surgery	82,840	Early discontinuation < 24 h vs. prophylaxis ≥ 24 h	SSI after spine surgery	Qualitative synthesis only
Inabathula et al., 2018 [14]	Retrospective before-and-after cohort	High-risk primary THA/TKA	2181	Standard prophylaxis vs. extended oral antibiotic prophylaxis	90-day PJI	Qualitative synthesis only
Flynn et al., 2024 [43]	Retrospective before-and-after cohort	Primary THA/TKA	4576	Extended oral antibiotic prophylaxis vs. standard prophylaxis	90-day infection after arthroplasty	Qualitative synthesis only
Bundschuh et al., 2024 [44]	Retrospective comparative cohort	Primary and aseptic revision THA/TKA	2982	Extended oral antibiotic prophylaxis vs. standard prophylaxis	3-month PJI	Qualitative synthesis only
Fuqua et al., 2025 [15]	Retrospective propensity-matched database cohort	Primary total hip arthroplasty	4153 receiving EOA	Extended oral antibiotic prophylaxis vs. no extended oral prophylaxis	PJI-free survivorship at 90 days, 1 year, and 2 years	Qualitative synthesis only
Fuqua et al., 2025 [16]	Retrospective propensity-matched database cohort	Primary total knee arthroplasty	5701 receiving EOA	Extended oral antibiotic prophylaxis vs. no extended oral prophylaxis	PJI-free survivorship at 90 days, 1 year, and 2 years	Qualitative synthesis only
Branch-Elliman et al., 2019 [18]	Multicentre national retrospective cohort	Cardiac, orthopaedic total joint replacement, colorectal, and vascular procedures	79,058	Increasing postoperative prophylaxis duration and prophylaxis type	SSI, AKI, and C. difficile infection	Qualitative safety synthesis
Carender et al., 2022 [19]	Retrospective cohort of patients who developed PJI	Primary or aseptic revision THA/TKA with subsequent PJI	119 PJI cases	Extended oral antibiotic prophylaxis vs. standard prophylaxis	Organism profile and antimicrobial resistance among PJI isolates	Qualitative resistance synthesis
Yang et al., 2025 [45]	Prospective cohort	Orthopaedic surgery; open fracture, closed fracture, and non-traumatic subgroups	37,046	Surgical antibiotic prophylaxis duration modeled as exposure	SSI within one year	Qualitative synthesis only
Virmani et al., 2024 [46]	Prospective observational study	Orthopaedic implant surgeries	264	Real-world antimicrobial prophylaxis regimens and durations	SSI, adherence to guidelines, adverse reactions, and costs	Qualitative implementation synthesis
Ferrer Pomares et al., 2025 [47]	Observational three-cohort study	High-risk instrumented spine surgery	132	Cefazolin 24 h vs. cefazolin plus amikacin 24 h vs. cefazolin plus amikacin 72 h	SSI at minimum 1-year follow-up	Qualitative synthesis only
Nagy et al., 2025 [48]	Retrospective cohort	Instrumented lumbar spine surgery	915	Cefazolin or clindamycin; single-dose vs. 72-h prophylaxis	SSI by antibiotic choice and duration	Qualitative synthesis only
Kweh et al., 2026 [49]	Retrospective single-centre cohort	Posterior instrumented spinal surgery	255	Standard 24-h postoperative prophylaxis vs. extended 2-week oral prophylaxis	Gram-positive SSI, return to theatre, complications, and infections	Qualitative synthesis only

**Table 2:** Leave-one-out sensitivity analysis for the primary surgical-site infection outcome. The pooled risk ratio ranged from 1.03 to 1.11 across all iterations, all 95% confidence intervals crossed the line of no effect, and I^2^ remained 0% in every analysis. These findings indicate that the overall interpretation was not dependent on any single trial.

Excluded Study	Pooled RR	95% CI	I^2^
Bhatnagar et al. 2017 [5]	1.08	0.87–1.35	0%
Hellbusch et al. 2008 [9]	1.05	0.84–1.32	0%
Takemoto et al. 2015 [6]	1.09	0.86–1.39	0%
Marimuthu et al. 2016 [10]	1.09	0.87–1.36	0%
Crist et al. 2018 [1]	1.03	0.82–1.30	0%
Urquhart et al. 2019 [7]	1.03	0.79–1.34	0%
Elsaqa et al. 2022 [11]	1.08	0.87–1.35	0%
PARITY Investigators 2022 [2]	1.05	0.79–1.38	0%
Nagata et al. 2022 [8]	1.11	0.88–1.40	0%
Ahmed et al. 2023 [12]	1.06	0.85–1.33	0%
Mathur et al. 2013 [31]	1.07	0.86–1.34	0%

**Table 3:** Summary of findings and GRADE certainty of evidence. **a.** Downgraded for risk of bias because several randomized trials had concerns regarding allocation reporting, open-label management, post-randomization exclusions, sparse events, or incomplete outcome reporting. **b.** Downgraded for imprecision because confidence intervals crossed the line of no effect and included clinically important benefit or harm. **c.** Eight trials reported deep or complicated SSI; one double-zero trial contributed to participant totals but not to the pooled risk-ratio estimate. **d.** Downgraded for indirectness because the evidence came from a single orthopaedic oncology trial. **e.** Two randomized trials reported Clostridioides difficile-associated colitis or pseudomembranous colitis. Nagata et al. reported zero events in both treatment groups and therefore contributed no statistical weight to the risk-ratio estimate. **Abbreviations:** CI, confidence interval; GRADE, Grading of Recommendations Assessment, Development and Evaluation; RR, risk ratio; SSI, surgical-site infection.

Outcome	Studies/ Participants	Relative Effect (95% CI)	Anticipated Absolute Effects	Certainty of Evidence	Interpretation
Any surgical-site infection	11 randomized trials/3823 participants	RR 1.07 (0.86–1.34)	Prolonged prophylaxis: 75 per 1000. Shorter prophylaxis: 80 per 1000 (65–101). Absolute difference: 5 more per 1000, ranging from 10 fewer to 26 more.	Low (a,b)	Prolonged prophylaxis did not demonstrate a statistically significant additional reduction in any SSI. The confidence interval remains compatible with possible benefit or harm and does not establish equivalence.
Superficial or non-deep SSI	7 randomized trials/1749 participants	RR 1.19 (0.79–1.77)	Prolonged prophylaxis: 47 per 1000. Shorter prophylaxis: 56 per 1000 (37–83). Absolute difference: 9 more per 1000, ranging from 10 fewer to 36 more.	Low (a,b)	No statistically significant difference was detected for superficial or non-deep SSI.
Deep or complicated SSI	8 randomized trials/2960 participants (c)	RR 0.85 (0.55–1.32)	Prolonged prophylaxis: 27 per 1000. Shorter prophylaxis: 23 per 1000 (15–36). Absolute difference: 4 fewer per 1000, ranging from 12 fewer to 9 more.	Low (a,b)	No statistically significant difference was detected for deep or complicated SSI.
Antibiotic-related complications	1 randomized trial/604 participants	RR 0.31 (0.12–0.85)	Prolonged prophylaxis: 51 per 1000. Shorter prophylaxis: 16 per 1000 (6–43). Absolute difference: 35 fewer per 1000, ranging from 45 fewer to 8 fewer.	Low (b,d)	Shorter prophylaxis was associated with fewer antibiotic-related complications in one orthopaedic oncology trial; generalizability to other procedures remains uncertain.
Healthcare-associated infection	1 cluster-randomized trial/1211 participants	RR 0.70 (0.38–1.27)	Prolonged prophylaxis: 66 per 1000. Shorter prophylaxis: 46 per 1000 (25–84). Absolute difference: 20 fewer per 1000, ranging from 41 fewer to 18 more.	Low (a,b)	No statistically significant difference was detected for healthcare-associated infection. The cluster-adjusted estimate was used.
Clostridioides difficile–associated colitis	2 randomized trials/1815 participants; 1 estimable effect (e)	RR 0.34 (95% CI 0.11–1.06)	Prolonged: 38 per 1000. Shorter: 13 per 1000 (4–40). Absolute difference: 25 fewer per 1000, ranging from 34 fewer to 2 more.	Low (b,d)	C. difficile-associated colitis was numerically less frequent with shorter prophylaxis, but the confidence interval crossed the line of no effect. The estimable effect was derived from one orthopaedic oncology trial.

## Data Availability

The data underlying this review are derived from published studies cited in the reference list. Study-selection decisions, extracted study characteristics, risk-of-bias judgments, and search strategies are available from the corresponding author upon reasonable request.

## Supplementary Materials

The following supporting information can be downloaded at: https://www.mdpi.com/article/10.3390/antibiotics15080759/s1, Supplementary Table S1: Complete electronic search strategies; Supplementary Table S2: SSI definitions, follow-up, and ascertainment in randomized duration-comparison trials. References [1,2,5,6,7,8,9,10,11,12,31,32] are cited in the Supplementary Materials.

## Abbreviations

The following abbreviations are used in this manuscript:

**Abbreviation**	**Full term**
ACL	Anterior cruciate ligament
AKI	Acute kidney injury
CENTRAL	Cochrane Central Register of Controlled Trials
CI	Confidence interval
*C. difficile*	*Clostridioides difficile*
df	Degrees of freedom
EOA	Extended oral antibiotic prophylaxis
GRADE	Grading of Recommendations Assessment, Development and Evaluation
I^2^	I-squared heterogeneity statistic
LOS	Length of stay
ORIF	Open reduction and internal fixation
PJI	Periprosthetic joint infection
PRISMA	Preferred Reporting Items for Systematic Reviews and Meta-Analyses
PROSPERO	International Prospective Register of Systematic Reviews
RCT	Randomized controlled trial
RoB 2	Cochrane Risk of Bias 2 tool
ROBINS-I	Risk Of Bias In Non-randomized Studies of Interventions
RR	Risk ratio
SSI	Surgical-site infection
THA	Total hip arthroplasty
TKA	Total knee arthroplasty
τ^2^	Between-study variance
χ^2^	Chi-square statistic
