# Supplementary material for: Shorter Versus Prolonged Systemic Perioperative Antibiotic Prophylaxis in Clean Orthopedic Surgery: A Systematic Review and Meta-Analysis
Source: Antibiotics (Basel). 2026 Aug 7;15(8):759. doi: 10.3390/antibiotics15080759 (PMC13509453; doi:10.3390/antibiotics15080759)
Supplement: Supplementary file 1 [file antibiotics-15-00759-s001.zip › antibiotics-4452949-supplementary-rev.pdf]

Supplementary Table S2. SSI definitions, follow-up, and ascertainment in randomized duration-comparison trials

Study	Procedure / comparison	SSI definition	Follow-up used	Post-discharge ascertainment	Adjudication
Bhatnagar et al., 2017 [5]	Hip implant surgery; single preoperative dose vs 5 postoperative doses.	Study-defined SSI; purulent discharge with culture or compatible clinical/implant findings. Superficial and deep SSI reported.	Minimum 12 months; 12-month outcome used.	Day-14 review and periodic reassessment to 1 year; patients advised to report fever or wound soakage.	Follow-up surgeon blinded to allocation.
Hellbusch et al., 2008 [9]	Instrumented lumbar fusion; single dose vs 3 days IV plus 7 days oral antibiotics.	Superficial infection defined by redness, oedema, or drainage; no deep infections occurred.	Maximum follow-up NR; all infections occurred within 21 days.	Post-discharge schedule and ascertainment method NR.	Independent/blinded adjudication NR.
Takemoto et al., 2015 [6]	Thoracolumbar spine surgery with drain; 24 hours vs antibiotics until drain removal.	CDC-defined SSI involving incision, fascia, or muscle; superficial and deep infection reported.	Minimum 1 year; 1-year outcome used.	Clinical review at postoperative visits; fewer than 10% followed by telephone.	Diagnosis by spine surgeon or infectious-diseases physician; surgeons blinded to duration.
Marimuthu et al., 2016 [10]	Instrumented spinal fusion; 24 vs 72 hours cefazolin.	CDC-described immediate/late SSI; superficial and deep infection reported.	At least 12 months for most patients; 12-month outcome used.	Post-discharge follow-up stated, but visit schedule and method NR.	Independent/blinded adjudication NR.
Crist et al., 2018 [1]	ORIF of closed fractures; no postoperative antibiotic vs 23-hour cefazolin.	Purulence plus inflammatory signs; superficial infection treated nonoperatively and deep infection required surgery.	Followed until bony union; outcome through union used.	Visits at 10-21 days, 6 and 12 weeks, then every 6-8 weeks until union.	Patients and clinical evaluators blinded.
Urquhart et al., 2019 [7]	Posterior thoracolumbar surgery with drain; 24 vs 72 hours.	CDC superficial, deep, and organ/space SSI; complicated SSI was deep or organ/space.	12 months; 12-month outcome used.	Telephone screen at 2 weeks; visits at 6 weeks and 3, 6, and 12 months.	Open-label; no independent adjudication committee reported.
Elsaqa et al., 2022 [11]	Primary hip/knee arthroplasty; 1 vs 3 postoperative days.	Clinical signs supported by laboratory tests and culture; superficial and deep SSI reported; no formal standard stated.	3 months; 3-month outcome used.	Outpatient visits at 2 and 6 weeks and 3 months.	Independent/blinded adjudication NR.
PARITY Investigators, 2022 [2]	Bone-tumour endoprosthetic reconstruction; 1 vs 5 postoperative days.	CDC superficial incisional, deep incisional, or organ/space SSI.	12 months; 1-year outcome used.	Scheduled assessments at 2 and 6 weeks and 3, 6, 9, and 12 months.	Blinded central adjudication committee.
Nagata et al., 2022 [8]	Mixed clean orthopaedic surgery; <24 vs 24-48 hours.	SSI within 30 days using modified CDC definitions.	30-day infection window; cluster-adjusted estimate used.	Patient reporting plus physician assessment or telephone/postal survey.	Participants masked; treating physicians unmasked; no independent adjudication reported.
Ahmed et al., 2023 [12]	Hip-fracture surgery; 1 preoperative dose vs 3 total doses.	Clinical wound signs; no formal depth-specific definition reported.	28 days; day-28 outcome used.	Wound assessments on postoperative days 3, 14, and 28.	Primary investigator assessed outcomes; blinding NR.
Mathur et al., 2013 [31]	ORIF of closed limb fractures; 24 hours vs prolonged IV plus oral antibiotics.	Inflammatory signs with pus; superficial/deep classification used.	Fixed maximum NR; routine follow-up to 10-14 days, with later opportunistic review.	Daily inpatient review, wound checks at 24 and 72 hours, and suture-removal visit at 10-14 days.	Follow-up assessor blinded.
Jacob Júnior et al., 2016 [32]	Lumbar arthrodesis; 1 vs 5 days.	Deep infection below fascia counted; superficial dehiscence was not counted as SSI.	90 days; not pooled because the SSI event was not assigned to a treatment arm.	Assessment at discharge, 2 weeks, and 90 days.	Blinding/adjudication NR.
