# Supplementary material for: Shorter Versus Prolonged Systemic Perioperative Antibiotic Prophylaxis in Clean Orthopedic Surgery: A Systematic Review and Meta-Analysis
Source: Antibiotics (Basel). 2026 Aug 7;15(8):759. doi: 10.3390/antibiotics15080759 (PMC13509453; doi:10.3390/antibiotics15080759)
Supplement: Supplementary file 1 [file antibiotics-15-00759-s001.zip › antibiotics-4452949-supplementary.pdf]

Supplementary Table S1. Complete Electronic Search Strategies

Shorter Versus Prolonged Systemic Perioperative Antibiotic Prophylaxis in Clean Orthopaedic Surgery

Search date: 14 May 2026 **Coverage:** Database inception to 14 May 2026 **Restrictions:** No language or publication-date restrictions

Search summary

Source	Platform/collection	Records retrieved	Search date
PubMed/MEDLINE	PubMed	601	14 May 2026
Scopus	Scopus	198	14 May 2026
Cochrane CENTRAL	Cochrane Library, Trials	236	14 May 2026
**Total**		**1035**	

S1. PubMed/MEDLINE

Interface: PubMed **Records retrieved:** 601

```
(
  "Orthopedic Procedures"[Mesh]
  OR "Spinal Fusion"[Mesh]
  OR "Arthroplasty, Replacement"[Mesh]
  OR "Fracture Fixation, Internal"[Mesh]
  OR "Prosthesis Implantation"[Mesh]
  OR orthop*[tiab]
  OR spine[tiab]
  OR spinal[tiab]
  OR "spinal fusion"[tiab]
  OR "spine surgery"[tiab]
  OR arthroplast*[tiab]
  OR "joint replacement"[tiab]
  OR "hip replacement"[tiab]
  OR "knee replacement"[tiab]
  OR "hip surgery"[tiab]
  OR "fracture fixation"[tiab]
  OR "internal fixation"[tiab]
  OR osteosynthes*[tiab]
  OR endoprosthe*[tiab]
  OR "tumor prosthesis"[tiab]
  OR "tumour prosthesis"[tiab]
)
AND
(
  "Antibiotic Prophylaxis"[Mesh]
  OR
  (
    (antibiotic*[tiab] OR antimicrobial*[tiab] OR antibacterial*[tiab])
    AND
    (prophyl*[tiab] OR perioperativ*[tiab] OR postoperativ*[tiab])
  )
)
AND
(
  duration[tiab]
  OR regimen*[tiab]
```

```
  OR schedule*[tiab]
  OR discontinu*[tiab]
  OR postoperativ*[tiab]
  OR postdischarge[tiab]
  OR "post discharge"[tiab]
  OR "single dose"[tiab]
  OR single-dose[tiab]
  OR "multiple dose"[tiab]
  OR "multiple doses"[tiab]
  OR multiple-dose*[tiab]
  OR "short course"[tiab]
  OR short-course[tiab]
  OR prolong*[tiab]
  OR extend*[tiab]
  OR "24 hour"[tiab]
  OR "24 hours"[tiab]
  OR 24-hour[tiab]
  OR "48 hour"[tiab]
  OR "48 hours"[tiab]
  OR 48-hour[tiab]
  OR "72 hour"[tiab]
  OR "72 hours"[tiab]
  OR 72-hour[tiab]
  OR "drain removal"[tiab]
  OR "wound closure"[tiab]
)
AND
(
  randomized controlled trial[pt]
  OR controlled clinical trial[pt]
  OR random*[tiab]
  OR placebo[tiab]
  OR trial[tiab]
)
NOT
(
  animals[mh] NOT humans[mh]
)
```

S2. Scopus

Interface: Scopus Advanced Search **Records retrieved:** 198

```
TITLE-ABS-KEY(
  (
    orthop*
    OR spine
    OR spinal
    OR "spinal fusion"
    OR "spine surgery"
    OR arthroplast*
    OR "joint replacement"
    OR "hip replacement"
    OR "knee replacement"
    OR "hip surgery"
    OR "fracture fixation"
    OR "internal fixation"
```

```
    OR endoprosthe*
  )
  AND
  (
    (antibiotic* OR antimicrobial* OR antibacterial*)
    W/3
    (prophyl* OR perioperativ*)
  )
  AND
  (
    "single dose"
    OR "multiple dose"
    OR "multiple doses"
    OR "short course"
    OR "short duration"
    OR "extended prophylaxis"
    OR "prolonged prophylaxis"
    OR "postoperative prophylaxis"
    OR "postoperative antibiotics"
    OR "post-discharge antibiotics"
    OR "24 hour"
    OR "24 hours"
    OR "48 hour"
    OR "48 hours"
    OR "72 hour"
    OR "72 hours"
    OR "drain removal"
    OR (duration W/3 (antibiotic* OR antimicrobial* OR prophyl*))
  )
  AND
  (
    random*
    OR "controlled trial"
    OR "clinical trial"
    OR placebo
  )
)
```

S3. Cochrane Central Register of Controlled Trials (CENTRAL)

Interface: Cochrane Library Search Manager; results restricted to Trials **Records retrieved:** 236

#1 (orthop* OR spine OR spinal OR "spinal fusion" OR "spine surgery" OR arthroplast* OR "joint replacement" OR "hip replacement" OR "knee replacement" OR "fracture fixation" OR "internal fixation" OR endoprosthe*):ti,ab,kw

#2 (((antibiotic* OR antimicrobial* OR antibacterial*) NEAR/3 (prophyl* OR perioperativ*))):ti,ab,kw

#3 ("single dose" OR "multiple dose" OR "multiple doses" OR "short course" OR "short duration" OR "extended prophylaxis" OR "prolonged prophylaxis" OR "postoperative prophylaxis" OR "postoperative antibiotics" OR "post-discharge antibiotics" OR (24 NEXT hour*) OR (48 NEXT hour*) OR (72 NEXT hour*) OR "drain removal" OR (duration NEAR/3 (antibiotic* OR antimicrobial* OR prophyl*))):ti,ab,kw

#4 #1 AND #2 AND #3

Search documentation

- All database results were exported with full citation information for deduplication and screening.
- The number of records reported above represents the number retrieved before deduplication.
- The total number of records identified through database searching was 1,035.
